# Characteristics of Lawn and Garden Equipment Sound: A Community Pilot Study

**DOI:** 10.16966/2576-6430.106

**Published:** 2017-11-03

**Authors:** Erica Walker, Jamie L Banks

**Affiliations:** 1 Harvard T.H. Chan School of Public Health, Department of Environmental Health, 401 Park Drive, Landmark Center West, USA; 2 Quiet Communities, P.O. Box 533, Lincoln, MA, 01733, USA

**Keywords:** Noise, Lawn and garden equipment, Leaf blower, Community, Low frequency, Occupational health, Health effects

## Abstract

More than 11 million gas powered leaf blowers (GLB) operate in the US. Most are powered by inefficient 2-stroke engines. The noise is intolerable to some and many communities have enacted ordinances restricting their use. This pilot study aimed to characterize the low, medium, and high frequency sound components from commercial grade GLBs over distance, evaluate the adequacy of the A-weighted decibel (dB [A]) to describe this sound, and discuss the impact of GLB sound in a community setting. In a field experiment with 2 leaf blowers and a hose vacuum, the time averaged A-weighted dB(A) (LAEQ) and un-weighted dB (LEQ) metrics, and low, mid, and high frequency GLB sound components were evaluated at intervals out to 800 feet from point of operation (centroid). Sound levels at 50 feet for each GLB were consistent with manufacturer ratings. The sound from the 2 GLBs and hose vacuum exceeded 100 decibels for both LEQ and LAEQ at the centroid. At all distance intervals, LEQ was 11.2–12.5 decibels higher compared with LAEQ. With the exception of the high frequency dB, all sound metrics emitted from this equipment were found to exceed WHO outdoor daytime standards (55 dB) up to 800 feet away from the centroid. The dominance of the low frequency component over distance indicates that a dB-based metric is a more appropriate descriptor of this sound compared with a dB(A)-based metric. The loudness of GLB sound at point of operation is especially concerning for the auditory and non-auditory health of workers and others regularly exposed in close proximity. The ability of this sound - in particular its lower frequency components - to travel over long distances suggests that GLB sound has a wide ranging impact on surrounding communities and raises concerns over its adverse health impacts.

## Introduction

It is a common, yet flawed, assumption that noise (defined as unwanted sound) is an opportunity cost associated with technological advancement. This misconception holds especially true in the lawn and garden industry where, today, fuel-powered equipment is used to perform nearly all tasks once done manually. A commonly used piece of equipment in landscape maintenance is the commercial grade gas-powered leaf blower (GLB). Most GLBs used in the United States are powered with inefficient 2-stroke engines, housed in lightweight metal that offers little sound insulation, and run at three times the speed of an automobile engine (9000 rpm) producing a loud, monotonous sound [1]. Many people have described the sound as intolerable.

In 2011, it was estimated that more than 11 million GLBs were in operation in the United States [2,3]. GLBs are used to perform tasks ranging from leaf, dust and debris removal to gutter cleaning and snow removal around neighborhoods, schools, hospitals, and public spaces. Commercial-grade machines account for the majority of leaf blower activity [2]. According to manufacturer reports, the sound pressure levels of these machines exceed 95 A-weighted decibels (dB[A]) at the ear of the operator and typically 65–80 dB(A) at 50 feet. Comparing these levels to daytime sound standards set by the World Health Organization (WHO)-these levels are upwards of 15 dB(A) higher than the recommended 55 dB(A) [4]. Given its high sound level and frequent use in residential neighborhoods, schools, health care facilities, parks and other public spaces, it is not surprising that the GLB has come under attack.

Adverse health effects from sound include auditory effects such as hearing loss and tinnitus, and non auditory effects such as reduced cognitive performance and mental health, sleep disruption, ischemic heart disease, myocardial infarction, and hypertension [5]. Low frequency sound components are considered to have more severe adverse health effects compared with higher frequency components [6,7]. Adverse effects from sources of sound with low frequency components may occur at levels below 30 dB(A) [4]. Lower decibel standards are recommended for sources with low frequency components compared with other sources [4].

Aside from dB(A) ratings reported by manufacturers, little is known about the character of GLB sound or its propagation into the environment. Finnish researchers found low frequency and tonal components in tests of several GLB models sound [8]. These factors in combination with the ability of low frequency sound to travel long distances and penetrate building walls [6], may explain the intolerability of this sound source to people in the community.

Given that the A-weighting system de-emphasizes frequencies below 500 Hz, a dB(A) may not adequately represent GLB sound and its potential impacts. The goals of this pilot study were to: 1) characterize the sound of a typical combination of leaf blowers used in commonly in lawn and garden maintenance over distance; 2) evaluate the adequacy of the dB(A) as a representative metric of that sound; and 3) discuss the impacts this equipment may have on surrounding communities. A field experiment measured sound at intervals out to 800 feet using A-weighted and other sound metrics.

## Materials and Methods

### Area of study

**Figure 1:**
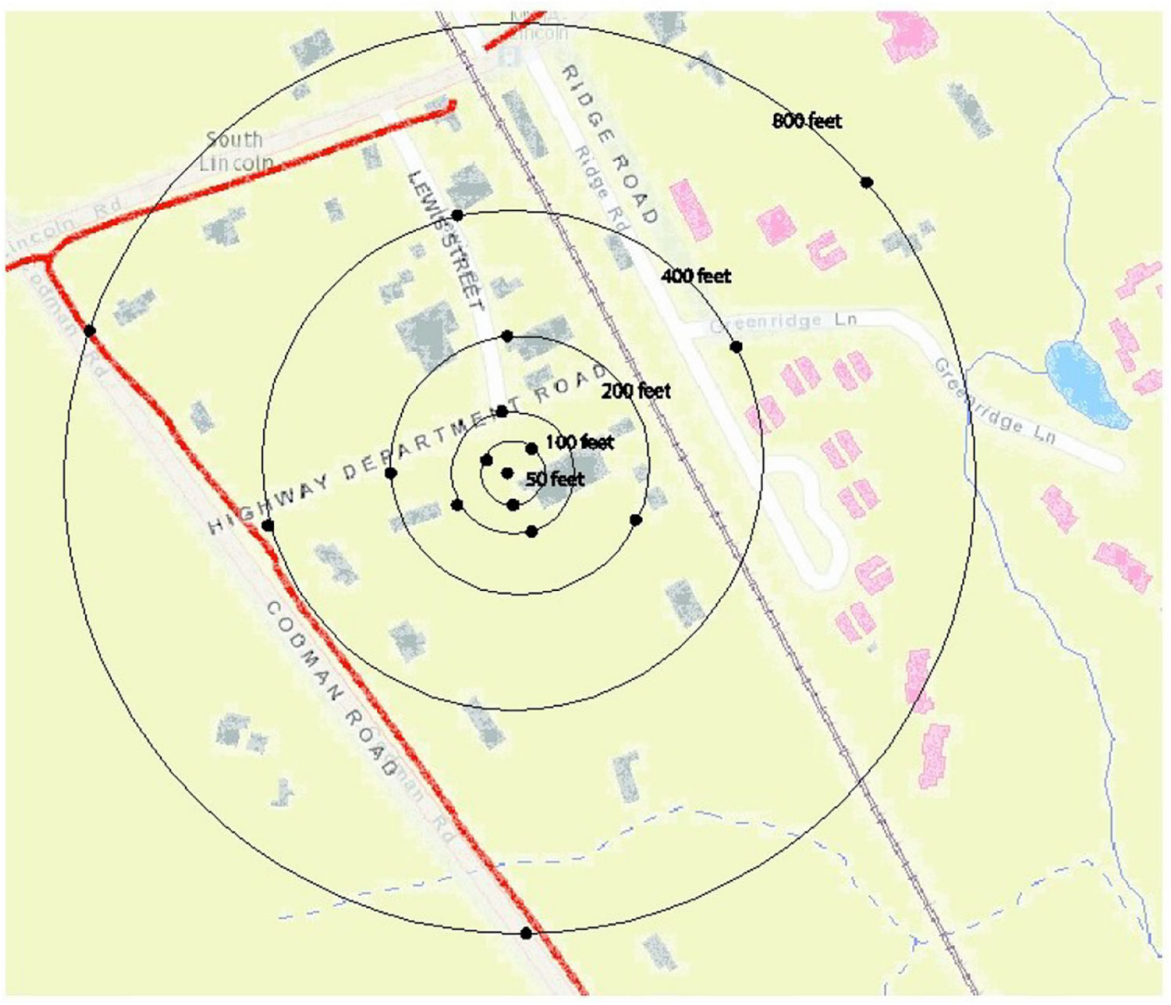
The 800 feet radius was delineated into concentric circles 50, 100, 200, 400, and 800 feet from the centroid

Field work was carried out on April 29, 2015, between the hours of 8 am and 11 am at the Department of Public Works (DPW) in Lincoln, MA, a suburb located approximately 20 miles from the City of Boston. Lincoln has a population of 6,500 and occupies a land area of 15 square miles. The study area’s starting point (distance 0, the “centroid”) was in the DPW parking lot and extended out to a radius of 800 feet (Figure 1). The 800 feet radius was delineated into concentric circles 50, 100, 200, 400, and 800 feet from the centroid.

Within the 800 feet radius were single and multifamily dwellings, as well as commercial and municipal properties. Light road, train, and aircraft traffic offered little to no competing sound.

### Lawn and garden equipment

Two backpack leaf blowers (EBZ8050 (machine “A”), and either the EB7000 (machine “B”) or the EBZ8001 (machine “C”) (Husqvarna Corporation, Stockholm, Sweden)) and one hose vacuum (machine “D”; Scag Giant-Vac, Mayville, WI) owned and operated by the DPW were used in this pilot study. Relevant specifications on these machines are detailed in Table 1. Sound from this equipment was measured as follows: (1) At 0 and 50 feet, each individual machine (A-D) was tested to compare observed sound levels to those reported by the manufacturer; (2). At 0, 50, 100, 200, 400, and 800 feet, a combination of three machines-two backpack leaf blowers (machines A at all distances and B (at distance 0 and 50) or C (at other distances) and the hose vacuum (machine D) were run simultaneously. This combination of equipment used commonly in today’s landscape maintenance practices. Beyond the centroid, for the combination scenario only, three measurements were taken at each distance. Machine C was used at distance intervals ≥100 feet because of a malfunction with machine B. Machine operators were asked to start the machines, bring them quickly from idle to full throttle, and remain at full throttle for 30–45 seconds.

### Sound and site condition measurement

An Optimus Red Octave Band Analyzer CR-162C (North Yorkshire, UK) was used to record sound pressure levels. Meteorological conditions (temperature, wind speed, relative humidity) were gathered using a Kestrel 3500 Weather Meter/Digital Psychrometer (Birmingham, MI, USA). Sound measurements were recorded for one minute. Efforts were made to avoid measuring the equipment sound in the presence of interfering sounds from trains, road traffic, aircraft, and emergency services. Measurements were repeated in the event of such interfering sounds.

### Sound metrics

The LAEQ and LEQ represent, respectively, the average dB(A) and dB sound pressures over the measurement period. For measurements at each interval distance, the LAEQ and LEQ were obtained directly from octave band analyzer. Low, medium, and high frequency sound metrics were calculated by summing the decibels across the following bands: low frequency (<250 Hz), medium frequency (250<Hz <2000), and high frequency (<2000 Hz). A background LAEQ was also measured at all distances to reflect the sound level when machines were not in operation.

### Data Analysis

For each distance (50, 100, 200, 400, 800 feet), the mean, standard deviation, and range were calculated taking the average of the three sound measurements at that distance. All analyses were conducted using SAS (version 9.4; SAS Institute Inc., Cary, N.C.).

## Results

Table 2 compares the observed sound levels at 50 feet to manufacturer rated sound levels at this same distance. Observed sound levels measured 77.6 – 80 dB(A) and were generally consistent with those reported by the manufacturers. At the centroid, the sound from the individual machines ranged from 85 – 101 dB(A) and 92–104 dB.

**Figure 2:**
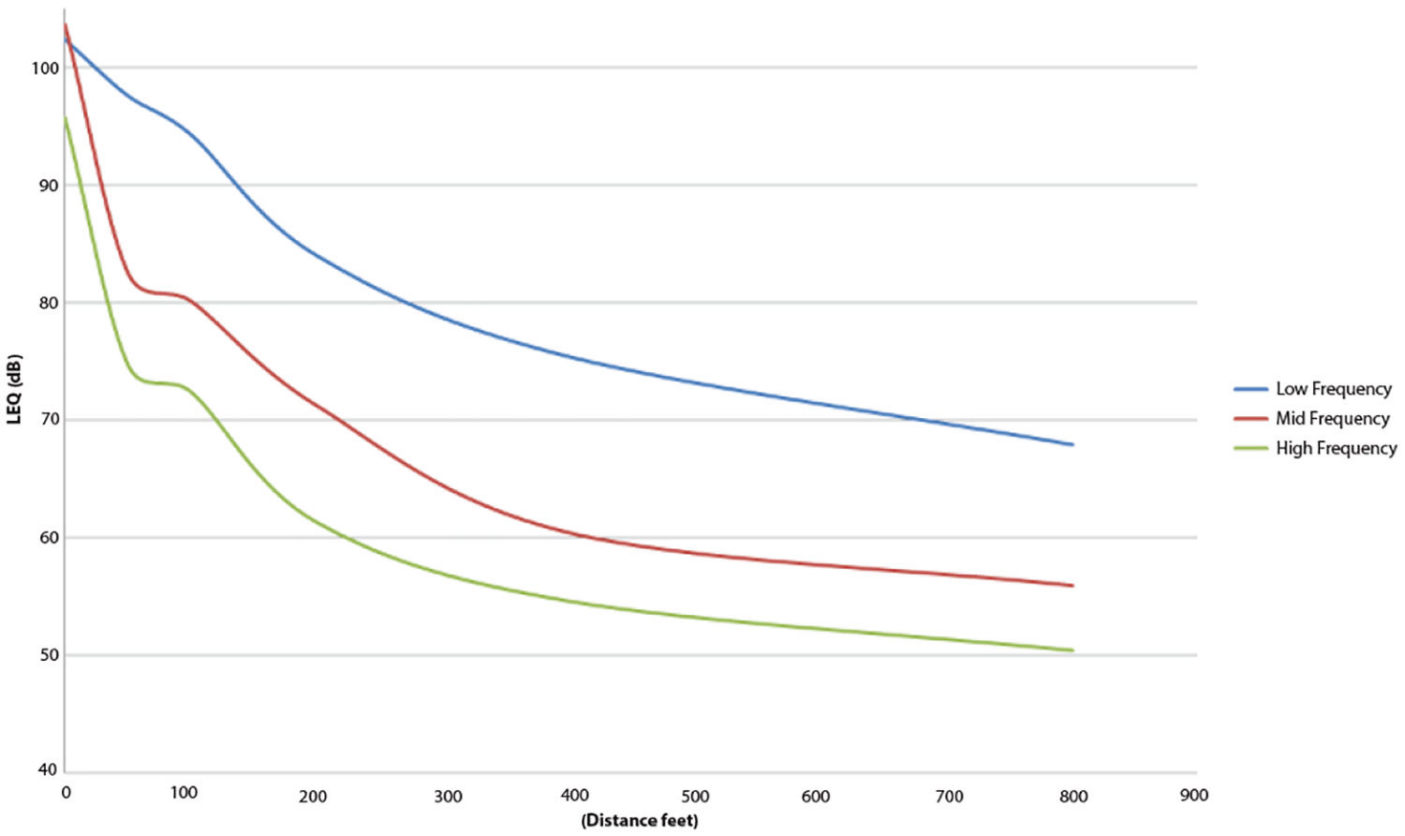
Sounds levels by frequency over distance

Descriptive statistics for sound emitted for the combination of machines at each distance are presented in Table 3. For all metrics, the highest sound levels occurred at the point of operation and decreased with distance. The difference between the LEQ and LAEQ range from 11.2–12.5 decibels at all distances. With the exception of the high frequency component, all sound metrics remain above recommended WHO outdoor daytime levels of 55 dB out to 800 feet. Low and mid-frequency were the dominant sound frequencies at all distances. Figure 2 displays the propagation of these frequencies out to 800 feet. Mid and low frequency sound levels dropped sharply within 100 feet from the centroid, while low frequency sound levels decreased more gradually over distance. At 800 feet compared to the centroid, the decrease in sound pressure was 45 dB(A) for LAEQ, 45 dB for the high frequency component, 51 dB for the mid frequency component, and 35 dB for the low frequency component.

## Discussion

Our results show that according to the most commonly used dB(A)-based metrics, lawn and garden equipment sounds were higher than the WHO’s community outdoor daytime sound standards up to 800 feet away from the centroid. When moving beyond the LAEQ to consider the full frequency profile of this type of sound, low frequency sound dominated at all distances and at very high decibels, consistent with results previously reported [8].

The finding that lawn and garden equipment sound remains higher than WHO outdoor daytime sound level standards of 55 dB(A), out to 800 feet from the point of operation, raises concerns regarding impact on communities. First, in this pilot study, we only considered the impact of a single typical lawn and garden maintenance operation. The reality is that within a given community, several operations may be occurring simultaneously and continue over prolonged periods of time-intensifying the harmful effects of these sound sources. Second, the area encompassed by a 1600-foot diameter circle (800-foot radius) is large. In a densely populated community, people who work from home, people who work night shifts, children, the retired, the elderly, and the sick may be exposed to high level low frequency sound in their homes, apartment complexes, and businesses. Additionally, this area may include people in schools, hospitals, daycare centers, and retirement homes for whom WHO daytime sound standards are 35 dB(A) or less [4].

In addition to the loudness of the sound emitted from these machines, the dominance of low frequency sound is concerning because of the ability of this sound to travel over long distances, penetrate construction walls and negatively impact health, productivity, and/or quality of life. According to previous studies, low-frequency sound is a common cause of annoyance and other stress-induced adverse [4,6,7]. Even at levels deemed to be non-harmful by the WHO, studies have shown that sources containing low frequency components gave rise to a multitude of complaints [6,9]. Beyond annoyance, low frequency sound is associated with acute changes in stress and cardiovascular responses such as: changes in blood pressure, heart rate variability, cortisol and amylase secretion and sleep disturbance [6]. Chronically, the continuous stimulation of these stress and cardiovascular responses can lead to hypertension, myocardial infarction, arteriosclerosis, ischemic heart disease, and stroke [5]. Regular exposure to GLB sound is likely to have negative effects on the auditory and non-auditory health of workers and others in close proximity.

The use of a dB(A)-based metric to represent sources of sound with strong low frequency components has been widely criticized [4,6,7,10]. The results of this pilot study suggest that A-weighted sound metrics are not adequate for representing the impact of sound from GLBs. Because low frequency sound travels extended distances with very little energy loss [6] and is known to penetrate construction walls [8] it is important for manufacturer ratings to include a frequency breakdown of the sound from their equipment and at distances beyond 50 feet. In addition, it is important for policy makers to understand the impact this type of sound may have on surrounding communities

To our knowledge, this is the first study to characterize landscape maintenance equipment sound over distance and in a community setting. The results provide a better understanding of the nature of the sound emitted from GLBs, and will be useful in informing regulatory and abatement policies within communities. However, there are several limitations to this pilot study which should be considered. Sound was only measured from a small number of machines at a single location over a short amount of time. In actual settings, many machines may operate on multiple properties within a neighborhood over the course of a day. Another limitation is that the study only measured ambient outdoor lawn and garden sound levels which may not reflect the sound levels experienced in indoor environments such as homes, offices, and schools. Additionally, our results are only descriptive and did not take into account sound propagation (attenuation or exacerbation due to physical or natural impedance) directly. Lastly, although the frequency spectrum augments our understanding of the nature of GLB sound beyond that offered by dB(A)-based metrics, the use of psychoacoustic metrics should be considered to help us to better understand the subjective human response to this type of sound [11].

Additional studies are needed to tackle these limitations. In particular, larger, more robust studies are needed to further investigate the interaction between factors such as prevalence and duration of use, time of day, location of exposure, distance from the source, sound propagation, and human response to better understand the impact of this sound on communities.

## Conclusions

The results of this study indicate that landscape maintenance sound produced by GLBs may travel over long distances in a community at levels known to increase the risk of adverse health effects. Vulnerable populations include workers, children, the elderly, the sick, those who work from home, and those who work overnight shifts. A-weighted decibel metrics do not adequately characterize GLB sound and its potential impact on a community. Because of the prevalent use of this type of equipment, it is important for health professionals and policy makers to understand the impact this type of sound may have on surrounding communities.

## Figures and Tables

**Table 1:** Summary of GLBs Used

Code	Engine Power	Max Air Flow (CFM)	Max air speed (MPH)
	(CC)		
A	72 cc	685	201
B	62 cc	529	200
C	72 cc	685	201

CC: cubic centimeters; CFM: cubic feet per minute; GLB: Gas powered leaf blower; MPH: miles per hour

**Table 2:** Manufacturer-Rated *vs* Observed Sound Levels of Equipment

Code	Manufacturer Rated Sound Level (dB[A] at 50’)	Observed Sound Level (dB[A] at 50’)
A	77	77.6
B	73	79
C	77	79
D	NA	80.6

**Table 3:** Sound Metrics for Two Leaf Blowers and a Hose Vacuum By Distance (dB)*

Distance (in feet)	Machine Combination Tested	Sound Metric	Mean (SD)	Range**
0	A+B+D	Background dB(A)	64.2	
		LEQ dB	106.4	
		LAEQ dB(A)	102.4	
		Low Frequency dB	102.4	
		Mid Frequency dB	103.6	
		High Frequency dB	95.7	
50	A+B+D	Background dB(A)	59.7	
		LEQ dB	97.6 (7.1)	87.3 – 101.3
		LAEQ dB(A)	85.5 (7.6)	74.0 – 88.5
		Low Frequency dB	97.6 (7.1)	87.2 – 101.2
		Mid Frequency dB	82.5 (7.2)	71.6 – 84.6
		High Frequency dB	74.7 (6.7)	64.6 – 77.5
100	A+C+D	Background dB(A)	49.7	
		LEQ dB	94.3 (5.5)	86.7 – 97.6
		LAEQ dB(A)	82.8 (7.0)	72.3 – 85.4
		Low Frequency dB	94.3 (5.4)	86.7 – 97.4
		Mid Frequency dB	80.1 (9.4)	65.5 – 82.1
		High Frequency dB	72.4 (10.4)	56.1 – 74.5
200	A+C+D	Background dB(A)	47.6	
		LEQ dB	85.2 (7.6)	72.9 – 87.9
		LAEQ dB(A)	72.9 (8.3)	60.5 – 77.0
		Low Frequency dB	84.0 (7.5)	72.6 – 87.6
		Mid Frequency dB	71.2 (9.2)	57.9 – 76.2
		High Frequency dB	61.3 (8.4)	48.5 – 61.1
400	A+C+D	Background dB(A)	58.3	
		LEQ dB	75.8 (1.1)	74.3 – 76.5
		LAEQ dB(A)	63.3 (2.2)	60.5 – 64.9
		Low Frequency dB	75.4 (1.0)	74.3 – 76.3
		Mid Frequency dB	60.4 (3.7)	55.3 – 62.1
		High Frequency dB	54.6 (6.3)	47.1 – 58.7
800	A+C+D	Background dB(A)	54.7	
		LEQ	68.5 (2.6)	67.7 – 70.6
		LAEQ dB(A)	57.3 (5.0)	50.7 – 60.6
		Low Frequency dB	67.9 (2.3)	65.6 – 70.5
		Mid Frequency dB	55.9 (7.3)	44.9 – 59.5
		High Frequency dB	50.4 (7.2)	39.7 – 54.0

* LAEQ is in dB(A);

** Range: the minimum and maximum values obtained over 3 recordings at each distance interval

LF: Low frequency (<250 Hz); MF: medium frequency (250<Hz<2000 Hz); HF: high frequency (<2000 Hz)
